# Next-Generation Sequencing Improves Diagnosis, Prognosis and Clinical Management of Myeloid Neoplasms

**DOI:** 10.3390/cancers11091364

**Published:** 2019-09-13

**Authors:** Diego Carbonell, Julia Suárez-González, María Chicano, Cristina Andrés-Zayas, Juan Carlos Triviño, Gabriela Rodríguez-Macías, Mariana Bastos-Oreiro, Patricia Font, Mónica Ballesteros, Paula Muñiz, Pascual Balsalobre, Mi Kwon, Javier Anguita, José Luis Díez-Martín, Ismael Buño, Carolina Martínez-Laperche

**Affiliations:** 1 Department of Hematology, Gregorio Marañón General University Hospital, 28007 Madrid, Spain; diego.carbonell@iisgm.com (D.C.); maria.chicano@salud.madrid.org (M.C.); gabriela.rodriguez@salud.madrid.org (G.R.-M.); marianabeatriz.bastos@salud.madrid.org (M.B.-O.); patricia.font@salud.madrid.org (P.F.); monica.ballesteros@salud.madrid.org (M.B.); paula.muniz@iisgm.com (P.M.); pascual.balsalobre@salud.madrid.org (P.B.); mi.kwon@salud.madrid.org (M.K.); javier.anguita@salud.madrid.org (J.A.); jdiezm@salud.madrid.org (J.L.D.-M.); ismaelbuno@iisgm.com (I.B.); 2 Gregorio Marañón Health Research Institute (IiSGM), 28007 Madrid, Spain; julia.suarez@iisgm.com (J.S.-G.); cristina.andres@iisgm.com (C.A.-Z.); 3 Genomics Unit, Gregorio Marañón General University Hospital, IiSGM, 28007 Madrid, Spain; 4 Sistemas Genómicos, 46980 Valencia, Spain; jc.trivino@sistemasgenomicos.com; 5 Department of Medicine, School of Medicine, Complutense University of Madrid, 28040 Madrid, Spain

**Keywords:** Next-generation sequencing, myeloid neoplasm, routine diagnosis, acute myeloid leukemia

## Abstract

Molecular diagnosis of myeloid neoplasms (MN) is based on the detection of multiple genetic alterations using various techniques. Next-generation sequencing (NGS) has been proved as a useful method for analyzing many genes simultaneously. In this context, we analyzed diagnostic samples from 121 patients affected by MN and ten relapse samples from a subset of acute myeloid leukemia patients using two enrichment-capture NGS gene panels. Pathogenicity classification of variants was enhanced by the development and application of a custom onco-hematology score. A total of 278 pathogenic variants were detected in 84% of patients. For structural alterations, 82% of those identified by cytogenetics were detected by NGS, 25 of 31 copy number variants and three out of three translocations. The detection of variants using NGS changed the diagnosis of seven patients and the prognosis of 15 patients and enabled us to identify 44 suitable candidates for clinical trials. Regarding AML, six of the ten relapsed patients lost or gained variants, comparing with diagnostic samples. In conclusion, the use of NGS panels in MN improves genetic characterization of the disease compared with conventional methods, thus demonstrating its potential clinical utility in routine clinical testing. This approach leads to better-adjusted treatments for each patient.

## 1. Introduction

Myeloid neoplasms (MN) constitute a group of hematological diseases that include acute myeloid leukemia (AML), myelodysplastic syndromes (MDS), myeloproliferative neoplasms (MPN) and myelodysplastic syndromes/myeloproliferative neoplasms (MDS/MPN) [1]. The genetic profile of these diseases is heterogeneous, and the number of alterations to be analyzed for proper risk stratification and management of affected patients increases constantly [2,3,4,5]. Moreover, the development of specific pathway-targeting treatments in combination with advances in genomic biomarker discovery is paving the way for precision medicine for many patients [6,7,8]. Consequently, current clinical testing attempts to include multiple molecular (e.g., Sanger sequencing, qPCR) and cytogenetic techniques (e.g., karyotype, FISH) with the aim of detecting a wide variety of genetic alterations. Next-generation sequencing (NGS) has become a useful tool to complete characterization of the spectrum of genetic variants in MN [5,9,10,11]. Nowadays, myeloid NGS panels can be applied to perform a complete analysis of genetic alterations in a single approach. These alterations are mainly single-nucleotide variants and small insertions and deletions, although some myeloid gene panels also cover copy number variants (CNV) and translocations. Although NGS myeloid panels are being implemented in routine diagnosis, there is no standard approach; therefore, features such as gene selection, sequencing platform, read depth and variant analysis can differ between laboratories. Moreover, myeloid panels are not usually designed to detect large alterations, mainly reported in whole genome NGS studies, which main limitation are their low depth and the difficulty to include them in daily clinical routine [12,13,14,15,16]. A clinically useful myeloid panel should have several characteristics to ensure optimal use of genetic data and subsequent processing. Gene selection is an important issue that should be based on a thorough review of the literature. In the last few years, new gene functional groups (up to nine in total) have been described in MN [17] Furthermore, clinical myeloid panels must include genes that are potentially “actionable”, i.e., genes whose variants are implicated in diagnosis, prognosis, treatment, and follow-up [18,19]. Additionally, germline variants in predisposition genes constitute another important group that was added in the 2016 revision of the WHO classification of MN [1] and should also be taken into account.

Several algorithms are used to classify variants into different categories according to their potential pathogenicity, and most of them focus on germline variants [19,20,21]. However, variants of uncertain significance (VUS) are a common finding during NGS analysis. Thus, VUS and their high frequency are significant issues that lead to incomplete and ambiguous reports and hence must be clarified [22,23].

Variant allele frequency (VAF) makes it possible to determine the clonality present in a diagnostic sample and to infer the variant subclonality based on the order of occurrence of each variant. In addition, the determination of the VAF for an individual variant and its comparison with that in subsequent samples have proven useful when evaluating clonal evolution [24,25,26,27]. Thus, the genetic study of samples at relapse provides information on which variants might be sensitive or resistant to treatment.

In this context, we evaluated the use of two enrichment-capture gene panels for the detection of gene variants, CNV and translocations that are frequently present in MN. In addition, we assessed the utility of analyzing paired samples at diagnosis and at relapse of AML in order to identify distinct patterns of clonal evolution in the natural history of the disease [25]. Thus, our main objective was to demonstrate the applicability of NGS as an analytical method in a clinical molecular laboratory and its clinical utility for improving the diagnosis, prognosis and treatment of MN.

## 2. Results

### 2.1. Variant Classification

**Figure 1:**
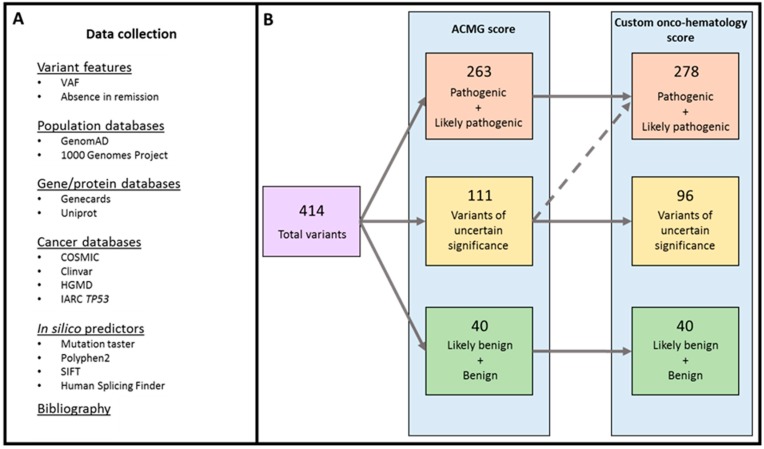
Algorithm for classification of variant pathogenicity. (**A**): Multiple databases were consulted. (**B**): The information was introduced in the American College of Medical Genetics and Genomics (ACMG) score, and an output with grade of pathogenicity was generated. Variants of uncertain significance (VUS) were reanalyzed using the onco-hematology score. The critical feature to classify them as pathogenic variant was their absence in remission in two cases, their variant allele frequency (VAF) values in seven cases and both in six cases (Table S1A).

The analysis revealed 414 variants in the 121 patients studied (Figure 1). Classification using the American College of Medical Genetics and Genomics (ACMG) score revealed 40 benign and likely benign variants, 111 VUS and 263 pathogenic and likely pathogenic variants (Table S1A–C). The custom onco-hematology score was applied to the 111 VUS, of which 15 were reclassified as pathogenic variants for a total of 278 (Table S1A). Of these reclassified variants, the critical features considered to classify them as a pathogenic variant was their absence in remission samples in two cases, their VAF values in seven cases and both features in six cases. Only pathogenic and likely pathogenic variants were included in the analysis.

### 2.2. Variant Distribution

**Figure 2:**
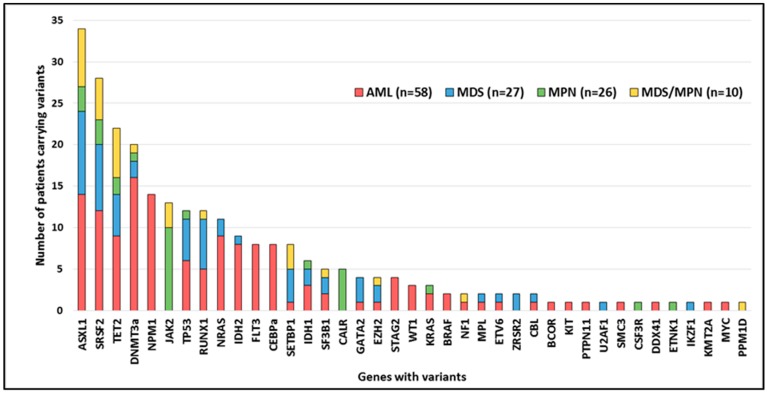
Number of patients carrying variants in each of the genes studied. AML: acute myeloid leukemia. MPN: myeloproliferative neoplasm. MDS: myelodysplastic syndrome. MDS/MPN: myelodysplastic syndrome/myeloproliferative neoplasm. Younger patients presented variants in *GATA2* (4 vs. 117 cases; 46 vs. 65 years; Mann–Whitney U test, *p* = 0.02), *CEBPa* (8 vs. 113 cases; 43 vs. 64 years; Mann–Whitney U test, *p* = 0.01) and *FLT3* (8 vs. 113 cases; 42 vs. 64 years; Mann–Whitney U test, *p* = 0.002). Patients affected by variants in *RUNX1* (12 vs. 109 cases; 69 vs. 63 years; Mann–Whitney U test, *p* = 0.04), *SETBP1* (8 vs. 113 cases; 73.5 vs. 63 years; Mann–Whitney U test, *p* = 0.03), *SRSF2* (28 vs. 93 cases; 72 vs. 61 years; Mann–Whitney U test, *p* << 0.001) and *ASXL1* (34 vs. 87 cases; 73 vs. 61 years; Mann–Whitney U test, *p* << 0.001) were older than those with the wild type. Compound heterozygosity was detected in patients in the following genes: *TET2* (*n* = 10), *CEBPa* (*n* = 3), *DNMT3a* (*n* = 2), *TP53* (*n* = 2), *WT1* (*n* = 1) and *STAG2* (*n* = 1). Additionally, triple heterozygosity in *TET2* was found in one patient.

At least one pathogenic variant was detected in 84% of patients (102/121), as follows: 91% (53/58) in AML, 85% (23/27) in MDS, 62% (16/26) in MPN (67% (2/3) in PV, 86% (6/7) in PMF, 46% (6/13) in ET and 67% (2/3) in MPN-unclassifiable) and 100% (10/10) in MDS/MPN. The number of variants per patient is shown in Figure S1. The most frequently mutated genes were *DNMT3a*, *NPM1*, and *ASXL1* in AML; *ASXL1*, *SRSF2*, and *RUNX1* in MDS; *JAK2* and *CALR* in MPN; and *ASXL1*, *TET2*, and *SRSF2* in MDS/MPN. The most ubiquitous mutated genes were *SRSF2* and *ASXL1*, which were found in each MN in at least 12% of patients (Figure 2). In addition, highly strong co-occurrence associations (Fisher test, *p* ≤ 0.0001) were found for variants in *ASXL1*-*SRSF2*, *ASXL1*-*SETBP1*, and *NPM1*-*DNMT3A*. Strong co-occurrence associations (Fisher test, *p* ≤ 0.01) were observed for variants in *IDH2*-*DNMT3A, SRSF2*-*TET2*, *NPM1*-*FLT3*, *SRSF2*-*RUNX1*, *FLT3*-*CEBPa*, *IDH2*-*STAG2*, and *ASXL1*-*RUNX1*. Conversely, variants in *NPM1*-*ASXL1* were mutually exclusive (Fisher test, *p* ≤ 0.01). Other associations (Fisher test, *p* ≤ 0.05) are shown in Figure S2.

### 2.3. Mutated Genes by Functional Group

**Figure 3:**
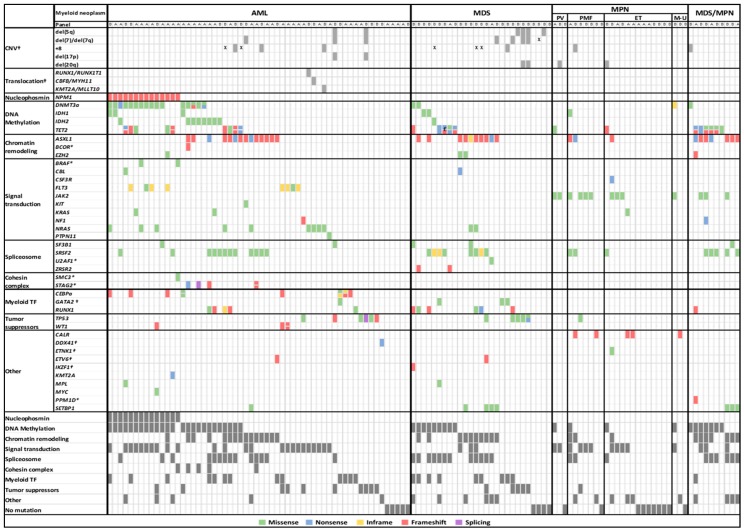
Variant landscape of diagnostic samples of 121 patients affected by myeloid neoplasms. Each column represents a patient. Colors represent the type of variant. *: Panel A exclusive genes (*BCOR*, *BCORL1*, *BRAF*, *UA2F1*, *SMC3*, *STAG2*, and *PPM1D*). *†*: Panel B exclusive genes and structural alterations (*GATA2*, *DDX41*, and *ETNK1*; CNV and translocations,). χ: CNV present detected by conventional methodologies but not detected by the NGS panel. £: This patient presented a triple variant in *TET2* (two nonsense and one frameshift). TF: transcription factor. AML: acute myeloid leukemia. MDS: myelodysplastic syndrome. MPN: myeloproliferative neoplasm. PV: polycythemia vera. PMF: primary myelofibrosis. ET: essential thrombocythemia. M-U: myeloproliferative neoplasm unclassifiable. MDS/MPN: myelodysplastic syndrome/myeloproliferative neoplasm. To determine the percentage of variants in genes studied exclusively by panel A (*BCOR*, *BCORL1*, *BRAF*, *U2AF1*, *SMC3*, *STAG2*, and *PPM1D*), the number of patients analyzed using this panel was considered as total (50 total MN, 37 AML, 2 MDS, 8 MPN, and 3 MDS/MPN). The same procedure was performed (71 total MN, 21 AML, 25 MDS, 18 MPN, and 7 MDS/MPN) for genes exclusively analyzed by panel B (*GATA2*, *DDX41*, and *ETNK1*; CNV and translocations).

*NPM1* was mutated in 12% of the cohort (14/121) and was detected only in AML patients (24%, 14/58). Moreover, most *NPM1*-mutated patients also presented variants in DNA methylation genes (93%, 13/14) and signal transduction genes (71%, 10/14; Figure 3). DNA methylation group genes were the most mutated among the whole cohort of patients (37%, 45/121). *DNMT3a* and *IDH2*, in particular, were more frequently mutated in AML than in the other MN (Figure 3). In the case of *DNMT3a* variants, 55% (12/22) were not the canonical p.R882 variant. Conversely, *TET2* was mutated in 18% of patients (22/121), and 50% of patients carried an additional variant in this gene (Figure 3). Signal transduction genes were mutated in 34% (41/121) of patients; in AML, the most frequently mutated genes within this group were *NRAS* (16%, 9/58) and *FLT3* (14%, 8/58; 6 ITD and 2 TKD). In the AML cohort, mutated signal transduction genes were mutually exclusive, with the exception of one patient who presented variants in *BRAF* and *NRAS*. In MDS, only 11% (3/27) of patients had mutated genes in this functional group. The most frequently mutated gene in MPN was *JAK2* (38%, 10/26). As for MDS/MPN, three patients had variants affecting signal transduction genes (Figure 3). In respect to chromatin-remodeling group, *ASXL1* was the most frequently mutated gene, and except for one patient who had variants in *EZH2*, patients with this functional group affected were not found in the *NPM1* AML subgroup (Figure 3). Regarding spliceosome group, *SRSF2* was the most frequently mutated gene. The variants detected were missense (affecting hotspot p.P95), whereas in MDS (30%, 8/27), inframe variants were also found (Figure 3). Variants in the cohesin complex group were a minority (10%, 5/50) because they were analyzed exclusively with panel A, found only in AML patients (14%, 5/37) and mainly affected *STAG2* (4/5) (Figure 3). 

Myeloid transcription factor variants were detected in all neoplasms except MPN. Variants in *CEBPa* were found exclusively in AML (14%, 8/58). *RUNX1* variants, which were predominantly truncating, were found in all MN except in MPN. All *GATA2* variants were missense and only present in AML and MDS patients (Figure 3). Variants in tumor suppressor genes were found in 16% (9/58) of AML patients, 19% (5/27) of MDS patients and 4% (1/26) of MPN patients. In this cohort, all *WT1* variants (2% 3/121) were frameshift and were found in AML (5%, 3/58) and always accompanied by a signal transduction variant. *TP53* variants were detected in 10% of patients (12/121), mainly in MDS (19%, 5/27), although they were also present in AML (10%, 6/58) and MPN (4%, 1/26; Figure 3).

### 2.4. Germline_Variants

In order to identify germline variants, 44 variants in six different genes (*CEBPa*, *GATA2*, *RUNX1*, *TP53*, *DDX41*, and *ETV6*) were checked, and 23/44 with VAF lower than 0.4 were ruled out, since they were not considered compatible with a germline origin. Of the remaining 21 variants, 17 were analyzed in a control sample from the same patient; nine were analyzed in cultured fibroblasts and eight in a blood sample at complete remission. No control samples were available for the remaining four cases. Finally, three variants were confirmed as germline: two cases of *GATA2* (GATA2 deficiency syndrome) and one case of *TP53* revealing a case of Li-Fraumeni syndrome (Table S2).

### 2.5. Structural and Numerical Alterations

Seventy-one patients were analyzed using panel B, which included CNV and translocations. Of the 34 alterations detected by cytogenetics, 28 were also detected by NGS in 25 patients. The three translocations were detected by both methods (*RUNX1*/*RUNX1T1*, *CBFB*/*MYH11*, and *KMT2A*/*MLLT10*), as were 25 of 31 CNV. The NGS panel did not detect six alterations identified by the karyotype: Five cases with trisomy of chromosome 8 and one case of monosomy of chromosome 7 (with low disease infiltration). On the other hand, NGS did detect alterations in several cases with low proliferation after the cell culture. Furthermore, in one case in which no metaphases were obtained, NGS was able to detect three different alterations: del(5q), del(7q), and del(17p) (Table S3).

### 2.6. NGS for Diagnosis, Prognosis, and Treatment Indication of MN

NGS revealed more genetic alterations than standard methodological approaches (Supplementary Information). At diagnosis, seven cases were reclassified by the use of NGS: Five as AML with mutated *RUNX1*, which were AML not otherwise specified (NOS) and two cases of MDS as MN with the germline *GATA2* variant (GATA2 deficiency syndrome). In respect to risk group classification, 15 cases of AML were reclassified from intermediate to adverse risk after detection of variants in genes *RUNX1*, *ASXL1*, and *TP53*. Prognosis of one patient affected by PMF was reclassified as adverse risk after the detection of variants in *ASXL1* and *SRSF2*. Finally, 44 patients were qualified to be included in clinical trials (22 AML, 13 MDS, three MPN, and six MDS/MPN), due to the presence of variants in *FLT3*-TKD, *IDH1*, *IDH2*, and spliceosome genes.

### 2.7. Clonality

**Figure 4:**
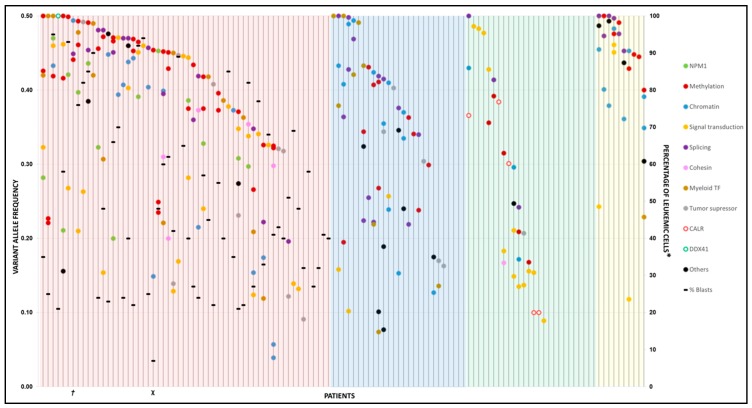
Variant spectrum of 121 myeloid neoplasms (MN) diagnosis samples. Each column represents a patient. Colored circles represent variants in genes classified by functional group and their corresponding VAF from 0 to 0.5. The background color represents the type of MN: acute myeloid leukemia (AML) (red), MDS (blue), MPN (green) and MDS/MPN (yellow). VAF was divided in half in case of deletion of the other allele. ***†***: Percentage of leukemic cells not available. **χ**: Peripheral blood sample. * Only in AML cases.

VAF was analyzed in 102 patients with at least one variant detected (Figure 4). In the AML cohort, the VAF distribution was highly heterogeneous, revealing the presence of several clones. Furthermore, the main variant (higher VAF) had a median VAF of 0.42 (range 0.09–0.5). The main variant affected the methylation machinery (32%), followed by signal transduction genes (17%). Interestingly, the VAF of genes involved in methylation, chromatin remodeling and splicing was greater than the blast percentage in most samples (81%, 42/52). Conversely, the VAF of variants that affect genes involved in proliferation and survival (signal transduction) was lower than the proportion of leukemic cells. In the 23 MDS, the median VAF of the main variant was 0.41 (range 0.16–0.5), and the main variant affected splicing-related genes (26%, 6/23). In the 16 MPN, the median VAF of the main variant was 0.31 (range 0.09–0.5), and the main variant affected the signal transduction gene group (38%, 6/16), particularly *JAK2*. Finally, for the ten MDS/MPN, the median VAF of the main variant was 0.41 (range 0.4–0.5).

### 2.8. Clonal Evolution

**Figure 5:**
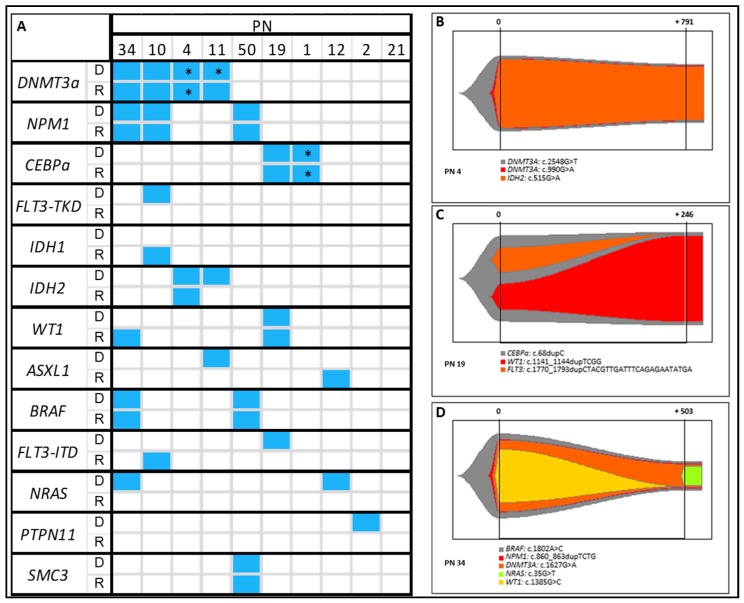
(**A**). Variants detected at diagnosis (D) and relapse (R) in ten relapsed AML patients. * Double variant. Examples of different patterns of clonal evolution in the cohort of relapsed AML. PN: patient number. (**B**). No change (PN 4), prevalence of clone identified at diagnosis. (**C**). Variant loss (PN 19) and (**D**). Both gain and loss of variants (PN 34), acquisition and loss of variants by the ancestral clone.

NGS analysis was performed in relapse samples of the ten relapsed AML patients, and the results were compared with the variant profiles of their respective diagnostic sample (Figure 5). Variants affecting *NPM1* (three out of three variants; 3/3), *CEBPa* (2/2), *BRAF* (2/2), and *SMC3* (1/1) at diagnosis were maintained at relapse in all cases. As a whole, 83% of variants in *DNMT3a* (5/6) were stable, except for one patient (PN 11) who had two *DNMT3a* variants at diagnosis and lost one at relapse. One of the two *IDH2* variants persisted at relapse. Finally, all variants in the juxtamembrane domain of *FLT3* (ITD) (0/1), tyrosine kinase domain of *FLT3* (0/1), *ASXL1* (0/1), *NRAS* (0/2) and *PTPN11* (0/1) were lost in relapse (Figure 5A). A new variant was acquired at relapse in *IDH1* (*n* = 1), *WT1* (*n* = 1), *ASXL1* (*n* = 1), and *FLT3*-ITD (*n* = 1) (Figure 5A). As for clonal evolution, three patterns were found in the study cohort: the same variants as those found at diagnosis (*n* = 4; Figure 5B), variant loss (*n* = 3; Figure 5C), and both loss and gain (*n* = 3; Figure 5D).

## 3. Discussion

The number of genetic variants that must be interrogated for an adequate clinical management of a particular patient has increased dramatically in recent years. Since conventional methods are insufficient to study all such variants, NGS is becoming the method of choice for an optimal genetic study. The benefits of using NGS panels in MN are not fully defined, and some areas remain unclear, namely, gene selection, ability to replace cytogenetic methods, clinical utility, classification of variants detected and the added value of NGS data (e.g., allele variant quantification and clonal evolution). Regarding other panels that are able to detect CNV and translocations, few have been reported and all of them are custom, meaning that they are not available for every laboratory [14,15,16]. In addition, panels with a large number of genes even exome analysis are usually applied in these studies, which are more expensive and require more samples for each sequencing run. Conversely, commercial standard panels, despite their benefits (bioinformatic analysis included, availability, validation, certification, etc.), do not usually include the detection of structural alterations [13]. In this study, we validated two commercial NGS panels in order to incorporate NGS in routine clinical testing. Thus, a NGS myeloid panel was used to interrogate a wide number of genes involved in MN. Then, as a result of the initial NGS experience, an optimized NGS myeloid panel was applied, which allows to detect variants in a lower, but more myeloid-specific number of genes (recently added to the 4th edition of the WHO classification [1]) and also structural alteration, improving variant detection. For these reasons, this commercial panel could be a good choice for routine analysis of myeloid neoplasms for every laboratory.

A total of 278 pathogenic variants were detected in 39 genes in 121 cases of MN. The number of genes altered and the most frequent variants in mutated genes in MN (also mutated genes by functional group), and the gene co-occurrences and mutual exclusivity were in accordance with previously results described in the literature [9,10,28,29,30,31,32,33,34,35,36,37,38,39,40,41,42]. In the case of MPN, there is a low percentage of patients carrying gene variants and the median number of variants per patient is low as well. This was probably due to the gene selection process, since authors who studied triple-negative MPN [43] found variants in atypical genes, which are not included in the panels used in the present study. Furthermore, it may be due to the presence of low VAF clones, which could be detected by increasing the read depth of sequencing. Regarding associations between mutated genes and age, variants affecting myeloid transcription factors (*GATA2* and *CEBPa*) as well as *FLT3* gene are prevalent in younger patients. On the contrary, variants affecting epigenetics or splicing related genes are more frequently found in elderly patients [44,45]. Germline gene variants and structural alterations enable a more thorough genetic analysis of MN. In our study, germline variants were found in three patients. Detection and validation of these alterations were essential to ensure optimal clinical management. In fact, in two allogeneic hematopoietic stem cell transplant (allo-HSCT) recipients, knowledge of the origin of the variant was crucial for donor selection. Genetic counseling for patients’ relatives is also important. Regarding structural alterations, translocations and CNV were detected in almost all patients. The case of undetected monosomy of chromosome 7 was due to the low infiltration of the sample, only detectable through FISH analysis of isolated CD34+ cells. In respect of trisomy of chromosome 8, NGS did not detect it in five out of eleven cases. This could be due to a proliferative advantage of the pathogenic clone, which may have been overrepresented after culture, and then could be detected by karyotype. Conversely, NGS was able to detect numerous alterations that have low number of altered metaphases in the karyotype and even when no metaphases were obtained in the cytogenetic study, thus making this approach useful in cases of low proliferating malignant clones. NGS is currently a complementary technique that must be further refined for the detection of both structural and germline alterations. However, this is, to the best of our knowledge, the first report of the usefulness of NGS in clinical routine for the detection of large structural alterations through a myeloid panel, instead of whole genome analysis.

Based on our results, NGS provides a more complete picture of gene variants than conventional methods. It allows to improve diagnosis, refine classification of prognosis and provide patients with tailored therapies (e.g., specific inhibitors). In our cohort, the results obtained with NGS made it possible to reclassify seven patients’ diagnosis and thus change management, especially in two cases reclassified as myeloid neoplasm with germ line predisposition from MDS. These two patients underwent allo-HSCT in order to replace their hematopoietic system, which is the treatment of choice for this type of myeloid neoplasm with germ line predisposition. Otherwise, without this reclassification, these patients would have remained untreated. As for prognosis, we were able to reclassify 15 patients as adverse risk from intermediate-risk, owing to the detection of variants in genes such as *RUNX1*, *ASXL1* (genes recently included in the ELN classification [2] and the GIPSS score [46]), and *TP53*. This reclassification of prognosis could be particularly relevant, since treatment for intermediate-risk patients is intensive chemotherapy regimen and allo-HSCT for adverse-risk patients. Clinical trials include specific myeloid gene inhibitors, such as *FLT3^inh^* (*midostaurin*) [47], *IDH1^inh^* (*ivosidenib*) [48], and *IDH2^inh^* (*enasidenib*) [49] or splicing *group^inh^* (*H3B-8800*) [50]. We retrospectively analyzed which patients could have been included in a clinical trial based on their gene variants. Thus, 44 patients could have been recruited for a clinical trial based solely on their variant profile. Moreover, NGS could prove useful for evaluating the response to specific therapies based on patient variant profile.

NGS also allows to determine the presence of clonal subpopulations and the sequence of variant acquisition by means of the VAF, thus making it possible to study clonal evolution. In this cohort, genes involved in splicing and epigenetics were the main groups of genes mutated in most patients. In the AML cohort in particular, the VAF of the variants was greater than the percentage of leukemic cells, thus supporting the hypothesis that the variants belong to the preleukemic clone. On the other hand, variants affecting genes involved in signal transduction pathways had a lower VAF than the proportion of leukemic cells in almost all cases [51]. In the MPN group, clones with variants in genes such as *JAK2* or *CALR* were the first to be acquired in most patients. Variants in other genes, like those involved in splicing and epigenetics may produce fewer damaging effects and may lead to the development of a slow progressing neoplasm, albeit generating susceptibility to the acquisition of additional variants [52]. Furthermore, variants affecting signal transduction genes, which are involved in proliferation pathways, lead to a faster progression. Finally, NGS analysis was performed in AML relapse samples, in order to evaluate the clonal evolution of the disease considering to the gain and loss of variants. In this regard, authors have postulated different clonal evolution patterns involving gain and loss of variants. Thus, clone detected at relapse could show the same variants as at diagnosis (AML initial clone), variant loss and gain (evolved ancestral clone) or change completely (unrelated clone) [25]. Variants that affect signal transduction genes and *ASXL1* have shown low stability, suggesting that cells with such variants are more sensitive to conventional treatment. As for acquisition, *IDH1*, *WT1*, *ASXL1*, and *FLT3*-ITD variants could be more likely to be gained at relapse or be present undetected at diagnosis and may be more resistant to chemotherapy than the other clones (Figure 5A) [24]. Regarding clonal evolution, we have found different patterns of loss and gain of variants at relapse. Four patients remained unchanged their variant profile at relapse. Cells harboring these variants were probably originated from the same linage as the clones identified at diagnosis, which could be more difficult to eradicate using conventional chemotherapy (Figure 5B). In the rest of cases variant gain or loss was observed. In these patients, a cell cluster harboring the founder variant could persist after chemotherapy, thus enabling a new expansion of an ancestral clone and generating susceptibility to acquisition of new variants in different genes (Figure 5C,D). Although NGS analysis provides useful information about the behavior of the disease, applying new approaches, such as single cell DNA and RNA sequencing, would allow a more comprehensive analysis of the effects of treatment in clonal evolution of AML [53,54]. In this aspect, non-genetic factors involved in drug resistance have been reported in AML relapsed patients, which could explain clonal evolution in these patients [55]. However, these methodologies are still far from being incorporated into clinical routine. Given that AML is a highly dynamic neoplasm, the analysis of relapse samples by NGS is essential to ensure optimal treatment.

## 4. Materials and Methods

### 4.1. Subjects Samples

The study population comprised 121 patients with a confirmed diagnosis of MN in our center between November 2015 and November 2018. AML and primary myelofibrosis (PFM) were included consecutively. Patients with MDS, polycythemia vera (PV), essential thrombocythemia (ET), and MDS/MPN were only included if they were considered suitable for therapy or included in a clinical trial; consequently, patients were not consecutive. The clinical characteristics of the study patients are shown in Table 1. Samples were obtained at diagnosis (107 bone marrow (BM) and 14 peripheral blood (PB) samples) from each of the 121 cases and at relapse in ten cases with AML (ten BM samples). The study was conducted in accordance with the Declaration of Helsinki, approved by the Ethics Committee of Gregorio Marañón General University Hospital, and all patients signed an informed consent. The 2016 WHO Classification of Tumours of Haematopoietic and Lymphoid Tissues was used to classify MN, and the ELN [2] and NCCN guides [56,57] as well as the GIPSS score [46] were used for classification of prognosis.

### 4.2. Targeted Sequencing and Variant Annotation 

Genomic DNA was purified from BM and PB samples using the *Maxwell RSC Blood DNA Kit* (*Promega*, Madison, WI, USA). Libraries were prepared using two enrichment-capture gene panels (*LMA-GeneSGKit* and *MyeloidNeoplasm-GeneSGKit*; Sistemas Genómicos, Valencia, Spain) according to the manufacturer’s protocol using 50 ng of DNA (Figure S3, Tables S4 and Table S5). Libraries were quantified using a *Qubit dsDNA HS Assay Kit* and a *Qubit 2.0* fluorimeter (Thermo Fisher, Waltham, MA, USA) and pooled in equal volumes. Paired-end sequencing (2 × 101 bp) was performed using the Illumina *MiSeq* platform (Illumina, San Diego, CA, USA). A mean depth of 700× was reached. FASTQ files were aligned against the human reference genome (version GRCh38/hg38) using the Burrows Wheeler Alignment tool [58] v0.7.15-r1140. Variant calling and indel-realignment were performed using a combination of two different algorithms: GATK [59] version 2.8–1 and VarScan [60] version 2.3.7. Integrative Genomics Viewer (Broad Institute, Cambridge, MA, USA) was used to visualize variants aligned against the reference genome to confirm the accuracy of the variant calls by checking for possible strand biases and sequencing errors. *Genesystems* software (*Sistemas Genómicos*, Valencia, Spain) was used in variant annotation in order to provide the infrastructure and interface for bioinformatic analysis (Supplementary Information).

### 4.3. Variant Analysis

Variants were classified into five levels of pathogenicity according to the American College of Medical Genetics and Genomics (ACMG) score [19]. Moreover, any VUS detected was reanalyzed with a custom onco-hematology score based on scores published elsewhere (Figure 1, Supplementary Information) [19,20,21]. Additional features such as VAF similar to other pathogenic accompanying variants and lack of the variants in a remission sample were taken into account in the custom score. The presence of the variants identified was confirmed using different techniques (Supplementary Information).

### 4.4. Statistical Analysis

Quantitative variables were expressed as median and range. Categorical variables were expressed as frequency and percentage. The Fisher exact test was used to compare the distribution of categorical variables. The Mann–Whitney test was used to compare differences between two independent variables. Statistical significance was set at *p* < 0.05 and all statistical test were two-sided. Analyses and fishplots were performed with R (3.3.2 version) [61].

## 5. Conclusions

To conclude, genetic analysis of MN samples through NGS panels is extremely useful for genetic characterization of patients and, therefore, for routine clinical testing. Diagnosis, prognosis, and treatment of MN could be improved with an accurate perspective of the variant landscape of the disease. Despite not being widely available, NGS panels are likely to be increasingly used in routine clinical testing.

## Figures and Tables

**Table 1:** Clinical characteristics of the 121 patients with myeloid neoplasms. AML: acute myeloid leukemia. MPN: myeloproliferative neoplasm. MDS: myelodysplastic syndrome. MN: myeloid neoplasm. WBC: white cell count. Hb: hemoglobin. HSCT: hematopoietic stem cell transplantation.

	Total	AML	MDS	MPN	MDS/MPN
**Patients (n)**	121	58	27	26	10
**Myeloid neoplasm**					
**AML**					
AML with recurrent genetic abnormalities	28	28	-	-	-
AML with myelodysplasia-related changes	25	25	-	-	-
AML, NOS	4	4	-	-	-
**MDS**					
MDS with excess blasts	14	-	14	-	-
MDS with multilineage dysplasia	4	-	4	-	-
MDS-RS and multilineage dysplasia	3	-	3	-	-
**MPN**					
Polycythemia vera	3	-	-	3	-
Primary myelofibrosis	7	-	-	7	-
Essential thrombocythemia	13	-	-	13	-
MPN, unclassifiable	3	-	-	3	-
**MDS/MPN**					
Chronic myelomonocytic leukemia	10	-	-	-	10
**MN with germline predisposition**	2	-	2	-	-
**Therapy-related MN**	5	1	4	-	-
**Age (median) (range)**	63 (23–86)	63 (23–86)	70 (40–82)	59 (27–78)	74 (50–80)
**Sex (female/male)**	(46/75)	(24/34)	(8/19)	(13/13)	(1/9)
**Blood count**					
WBC (× 10^9^/L)	6.3 (0.8–300.8)	6.2 (0.8–300.8)	4 (0.9–17.30)	7 (2.2–29.5)	11 (4.2–31.2)
Platelets (× 10^9^/L)	114 (11–1049)	74 (11–585)	70 (11–1001)	372 (114–1049)	116 (50–823)
Hb (g/L)	111 (53–171)	105 (53–143)	97 (59–146)	140 (103–171)	129 (99–153)
BM Blasts (%)	22 (0–95)	48 (20–95)	6 (0–19)	1 (0–9)	6 (1–17)
PB Blasts (%)	1 (0–98)	18 (0–98)	0 (0–6)	0 (0–7)	0 (0–2)
**LDH (U/L)-Normal range: 135–225**	258 (117–2504)	275 (123–2504)	218 (117–493)	233 (152–1534)	231 (131–447)
**Karyotype**					
Normal	48	26	8	9	5
Altered	43	21	17	2	3
Complex	7	6	1	0	0
No karyotype	23	5	1	15	2
**Treatment**					
Intensive chemotherapy	39	38	1	0	0
Hypomethylating agents	26	15	10	0	1
Hydroxyurea	10	0	0	8	2
Anagrelide	3	0	0	3	0
No treatment	43	5	16	15	7
**Allo-HSCT**	27	19	5	2	1
HLA identical	8	8	0	0	0
Haploidentical	19	11	5	2	1
**Relapse**	10	10	0	0	0
**Refractory**	11	11	0	0	0
**Progression to AML**	1	-	1	0	0

## Supplementary Materials

The following are available online at https://www.mdpi.com/2072-6694/11/9/1364/s1. Figure S1. Number of variants per patient with myeloid neoplasms. Figure S2. Pairwise associations between mutated genes. Figure S3. Genes included in NGS panels A and B. Table S1A. Pathogenic variants. Table S1B. Variants of uncertain significance. Table S1C. Benign variants detected. Table S2. Germline variants. Table S3. CNV and translocations. Table S4. Genes included in panel A. Table S5. Genes and alterations included in panel B. Supplementary Information.
