# Supplementary material for: Next-Generation Sequencing Improves Diagnosis, Prognosis and Clinical Management of Myeloid Neoplasms
Source: Cancers (Basel). 2019 Sep 13;11(9):1364. doi: 10.3390/cancers11091364 (PMC6770229; doi:10.3390/cancers11091364)
Supplement: Supplementary file 1 [file cancers-11-01364-s001.zip › cancers-588721-SI.pdf]

Supplementary Materials: Next-generation sequencing improves diagnosis, prognosis and clinical management of myeloid neoplasms.

Diego Carbonell, Julia Suárez-González, María Chicano, Cristina Andrés-Zayas, Juan Carlos Triviño, Gabriela Rodríguez-Macías, Mariana Bastos-Oreiro, Patricia Font, Mónica Ballesteros, Paula Muñiz, Pascual Balsalobre, Mi Kwon, Javier Anguita, José Luis Díez-Martín, Ismael Buño, Carolina Martínez-Laperche.

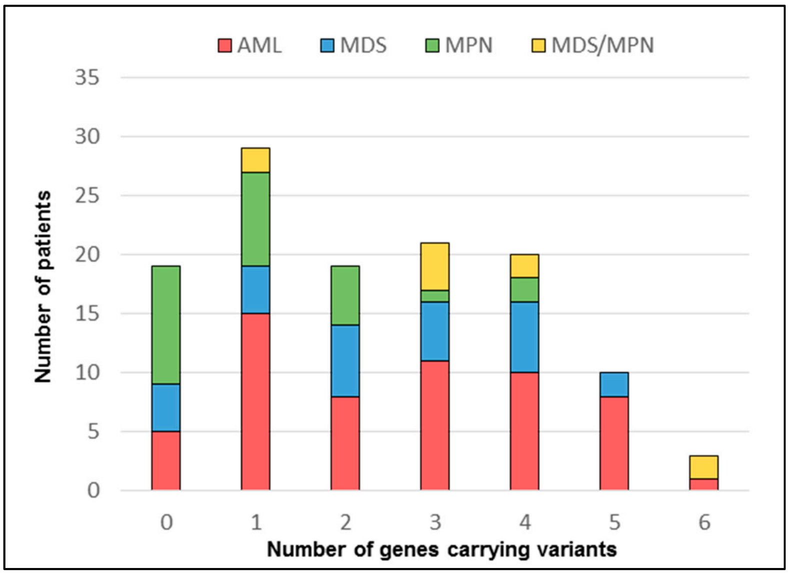

Figure S1. Number of variants per patient with myeloid neoplasms (n = 121). AML: acute myeloid leukemia. MPN: myeloproliferative neoplasm. MDS: myelodysplastic syndrome. MDS/MPN: myelodysplastic syndrome/myeloproliferative neoplasm.

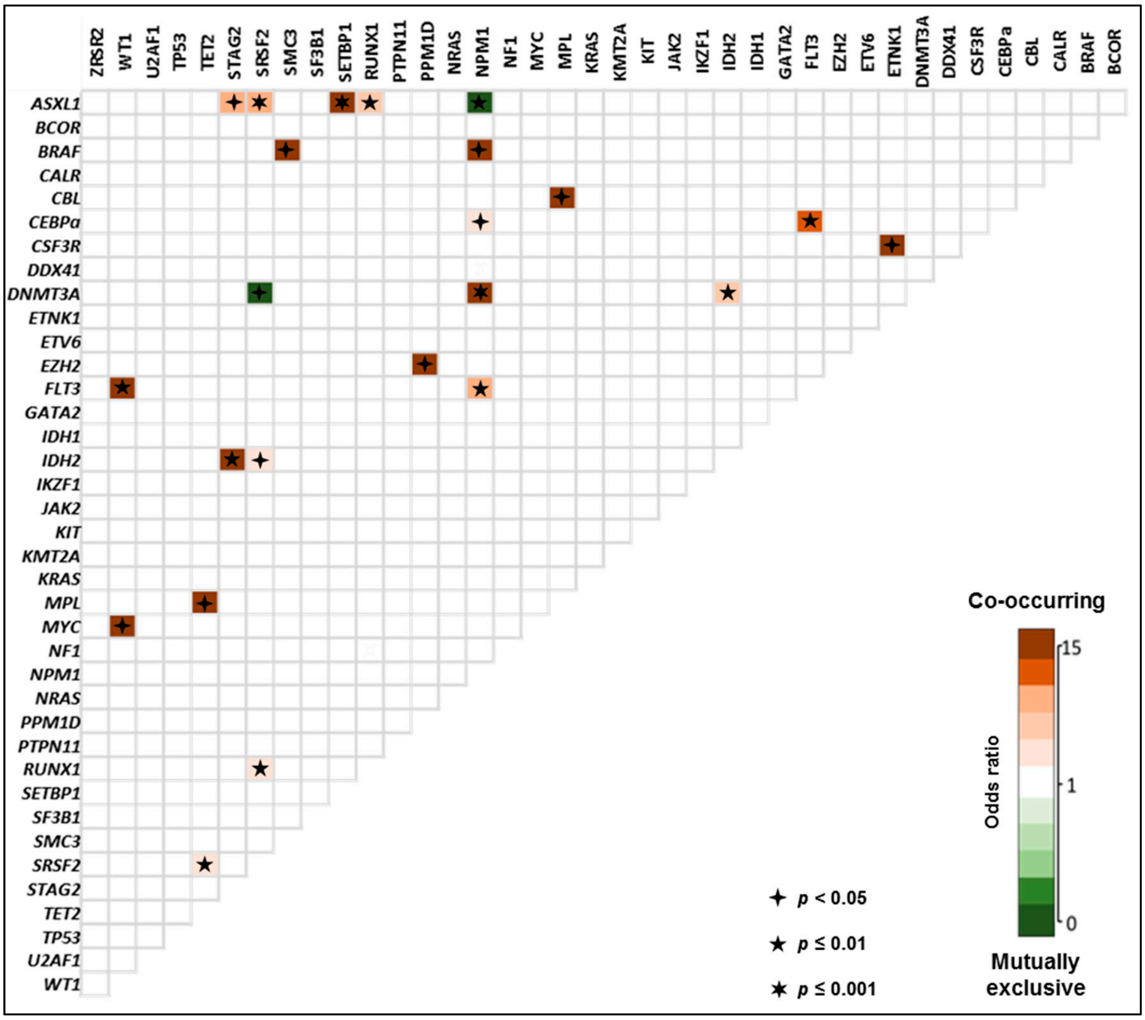

Figure S2. Pairwise associations between mutated genes.

NGS myeloid panel A and B genes					
ABL1	CNOT3	FLT3	KRAS	PPM1D	SRSF2
ANKRD26	CREBBP	GATA2	MAP1B	PTPN11	STAG1
ASXL1	CSF3R	GNAS	MPL	RUNX1	STAG2
BCOR	DDX41	IDH1	MYD88	SETBP1	TET2
BCORL1	DNMT3A	IDH2	NF1	SETD2	TP53
BRAF	EPOR	IKZF1	NOTCH1	SETDB1	U2AF1
CALR	ETNK1	JAK2	NPM1	SF3B1	VHL
CBL	ETV6	KIT	NRAS	SMC3	WT1
CEBPA	EZH2	KMT2A	PHF6	SOS1	ZRSR2

Figure S3. Genes included in NGS panels A and B. Blue, panel A exclusive genes; red, panel B exclusive genes; white, genes included in both panels. Germline predisposition genes are underlined. Panel A: indels and single-nucleotide variants (LMA-GeneSGKit; Sistemas Genómicos, Spain); panel B: indels, single-nucleotide variants, translocations, copy number variants (CNVs) and large numerical alterations (MyeloidNeoplasm-GeneSGKit; Sistemas Genómicos, Spain).

Table S1A. Pathogenic/likely pathogenic variants.

Gene	Variant	VAF	PN	
ASXL1	c.1609G>T	0.43	76	
ASXL1	c.1772dupA	0.494	116	
ASXL1	c.1774C>T	0.153	60	
ASXL1	c.1782C>A	0.391	48	
ASXL1	c.1900_1922delAGAGAGGCGGCCACCACTGCCAT	0.215	25	
ASXL1	c.1900_1922delAGAGAGGCGGCCACCACTGCCAT	0.172	56	
ASXL1	c.1900_1922delAGAGAGGCGGCCACCACTGCCAT	0.239	86	
ASXL1	c.1900_1922delAGAGAGGCGGCCACCACTGCCAT	0.149	92	
ASXL1	c.1900_1922delAGAGAGGCGGCCACCACTGCCAT	0.355	103	
ASXL1	c.1926dupA	0.424	73	
ASXL1	c.1934delG	0.443	11	
ASXL1	c.1934delG	0.039	15	
ASXL1	c.1934dupG	0.174	16	
ASXL1	c.1934dupG	0.373	33	
ASXL1	c.1934dupG	0.407	44	
ASXL1	c.1934dupG	0.361	47	
ASXL1	c.1934dupG	0.296	63	
ASXL1	c.1934dupG	0.473	64	
ASXL1	c.1934dupG	0.455	75	
ASXL1	c.1934dupG	0.154	82	
ASXL1	c.1934dupG	0.394	83	
ASXL1	c.1934dupG	0.433	95	
ASXL1	c.1934dupG	0.408	109	
ASXL1	c.1934dupG	0.379	111	
ASXL1	c.1934dupG	0.404	114	
ASXL1	c.1934dupG	0.448	117	
ASXL1	c.1934dupG	0.37	118	
ASXL1	c.2077C>T	0.401	78	
ASXL1	c.2309C>G	0.438	43	
ASXL1	c.2324delT	0.127	98	
ASXL1	c.2351delA	0.453	69	
ASXL1	c.2455G>T	0.494	102	
ASXL1	c.2734A>T	0.399	7	
ASXL1	c.2898_2900delAGG	0.489	94	
BCOR	c.2330dupC	0.057	15	
BRAF	c.1799T>A	0.338	50	
BRAF	c.1802A>C	0.444	34	
CALR	c.1092_1143del	0.1	58	
CALR	c.1092_1143del	0.1	85	
CALR	c.1127_1135GCAAGAGG>TTTGCTTA	0.301	104	
CALR	c.1135_1144delGAGGAGGAGG	0.366	76	
CALR	c.1154_1155insTTGTC	0.384	49	
CBL	c.1258C>T	0.257	86	
CBL	c.1259G>A	0.924	100	
CEBPa	c.178delA	0.496	6	
CEBPa	c.198_201dupCTAC	0.772	26	
CEBPa	c.247delC	0.418	1	
CEBPa	c.383delC	0.426	70	
CEBPa	c.59_60insTC	0.478	72	
CEBPa	c.622T>C	0.5	13	†
CEBPa	c.659T>C	0.5	13	†
CEBPa	c.68dupC	0.45	19	
CEBPa	c.934_936dupCAG	0.49	112	
CEBPa	c.944_945insCAC	0.392	1	*
CEBPa	c.971T>G	0.471	112	
CSF3R	c.2326C>T	0.149	63	
DDX41	c.931C>T	0.517	51	
DNMT3A	c.1031T>C	0.65	15	* †
DNMT3A	c.1627G>A	0.375	34	*
DNMT3A	c.1700_1702delTGG	0.168	77	*
DNMT3A	c.1733A>G	0.371	71	
DNMT3A	c.1913C>T	0.227	13	* †
DNMT3A	c.1920delT	0.436	11	
DNMT3A	c.2320G>A	0.453	11	* †
DNMT3A	c.2548G>T	0.361	4	
DNMT3A	c.2578T>C	0.411	89	
DNMT3A	c.2644C>T	0.429	28	
DNMT3A	c.2644C>T	0.456	57	
DNMT3A	c.2644C>T	0.426	70	
DNMT3A	c.2645G>A	0.465	10	

Table S1A (Cont'd)

Gene	Variant	VAF	PN	
DNMT3A	c.2645G>A	0.418	18	
DNMT3A	c.2645G>A	0.499	27	
DNMT3A	c.2645G>A	0.249	35	
DNMT3A	c.2645G>A	0.445	66	
DNMT3A	c.2645G>A	0.461	72	
DNMT3A	c.2645G>A	0.407	73	
DNMT3A	c.2705T>C	0.416	100	
DNMT3A	c.939G>A	0.466	59	
DNMT3A	c.990G>A	0.396	4	
ETNK1	c.734G>T	0.247	63	*
ETV6	c.306dupT	0.476	117	
ETV6	c.416_419delCTAT	0.077	103	
EZH2	c.1399delA	0.698	48	
EZH2	c.2023A>T	0.433	6	* †
EZH2	c.863G>A	0.41	86	*
EZH2	c.893G>A	0.67	118	*
FLT3	c.2503G>T	0.451	10	
FLT3	c.2503G>T	0.341	29	
FLT3	ITD	0.46	6	
FLT3	ITD	0.129	19	
FLT3	ITD	0.26	22	
FLT3	ITD	0.27	27	
FLT3	ITD	0.46	36	
FLT3	ITD	0.211	72	
GATA2	c.1186C>T	0.491	17	
GATA2	c.1187G>A	0.565	55	
GATA2	c.913C>G	0.433	24	* †
GATA2	c.989G>A	0.412	112	
IDH1	c.394C>G	0.299	107	
IDH1	c.394C>T	0.221	13	
IDH1	c.394C>T	0.235	35	
IDH1	c.394C>T	0.209	56	
IDH1	c.394C>T	0.195	109	
IDH1	c.395G>A	0.506	70	
IDH2	c.419G>A	0.452	7	
IDH2	c.419G>A	0.472	14	
IDH2	c.419G>A	0.322	15	
IDH2	c.419G>A	0.434	38	
IDH2	c.419G>A	0.363	54	
IDH2	c.419G>A	0.471	59	
IDH2	c.515G>A	0.373	4	
IDH2	c.515G>A	0.469	11	
IDH2	c.515G>A	0.451	28	
IKZF1	c.647_648delTA	0.101	89	
JAK2	c.1615A>T	0.428	53	
JAK2	c.1849G>T	0.483	3	
JAK2	c.1849G>T	0.183	37	
JAK2	c.1849G>T	0.135	56	
JAK2	c.1849G>T	0.211	63	
JAK2	c.1849G>T	0.451	64	
JAK2	c.1849G>T	0.971	68	
JAK2	c.1849G>T	0.118	69	
JAK2	c.1849G>T	0.243	75	
JAK2	c.1849G>T	0.156	77	
JAK2	c.1849G>T	0.089	88	
JAK2	c.1849G>T	0.477	93	
JAK2	c.1849G>T	0.137	113	
KIT	c.2447A>T	0.471	83	
KMT2A	c.1A>G	0.385	39	
KRAS	c.34G>A	0.154	58	
KRAS	c.35G>T	0.154	14	
KRAS	c.38G>A	0.24	18	
MPL	c.1771T>G	0.324	24	
MPL	c.1771T>G	0.156	100	
MYC	c.221C>T	0.274	71	
NF1	c.1621_1624dupATTG	0.169	40	
NF1	c.910C>T	0.451	64	
NPM1	c.860_863dupTCTG	0.47	6	
NPM1	c.860_863dupTCTG	0.328	18	
NPM1	c.860_863dupTCTG	0.421	27	

Table S1A (Cont'd)

Gene	Variant	VAF	PN	
NPM1	c.860_863dupTCTG	0.386	34	
NPM1	c.860_863dupTCTG	0.453	35	
NPM1	c.860_863dupTCTG	0.436	39	
NPM1	c.860_863dupTCTG	0.297	50	
NPM1	c.860_863dupTCTG	0.323	57	
NPM1	c.860_863dupTCTG	0.2	59	
NPM1	c.860_863dupTCTG	0.282	70	
NPM1	c.860_863dupTCTG	0.211	100	
NPM1	c.863_864insCATG	0.308	71	
NPM1	c.863_864insCATG	0.397	72	
NPM1	c.863_864insCCTG	0.391	10	
NRAS	c.181C>A	0.378	20	
NRAS	c.182A>G	0.446	41	
NRAS	c.34G>A	0.403	43	
NRAS	c.34G>A	0.132	119	
NRAS	c.34G>T	0.323	70	
NRAS	c.35G>A	0.124	82	
NRAS	c.35G>A	0.102	94	
NRAS	c.35G>T	0.282	34	
NRAS	c.38G>A	0.326	12	
NRAS	c.38G>A	0.158	95	
NRAS	c.38G>T	0.348	71	
PPM1D	c.1636dupC	0.304	48	
PTPN11	c.179G>T	0.139	2	
RUNX1	c.305dupT	0.527	109	
RUNX1	c.335dupT	0.074	89	
RUNX1	c.367_370dupGATG	0.307	14	
RUNX1	c.530_532delTCA	0.209	82	*
RUNX1	c.592G>A	0.363	8	
RUNX1	c.595G>A	0.219	73	
RUNX1	c.602G>A	0.379	95	
RUNX1	c.611G>A	0.221	7	
RUNX1	c.926dupG	0.119	16	
RUNX1	c.941_942delCT	0.229	48	
RUNX1	c.941_942delCT	0.136	105	
RUNX1	c.958C>T	0.421	116	
SETBP1	c.2602G>A	0.462	43	
SETBP1	c.2602G>A	0.175	98	
SETBP1	c.2602G>A	0.189	103	
SETBP1	c.2602G>A	0.24	118	
SETBP1	c.2602G>T	0.493	111	
SETBP1	c.2608G>A	0.437	47	
SETBP1	c.2609G>T	0.346	60	
SETBP1	c.2620G>A	0.487	75	
SF3B1	c.1997A>C	0.498	94	
SF3B1	c.1997A>G	0.481	57	
SF3B1	c.1998G>C	0.504	111	
SF3B1	c.2098A>G	0.419	89	
SF3B1	c.2098A>G	0.196	90	
SMC3	c.1973T>G	0.354	50	* †
SRSF2	c.281_283dupGCC	0.469	116	
SRSF2	c.284_307delCCCCGGACTCACACCACAGCCGCC	0.224	24	
SRSF2	c.284_307delCCCCGGACTCACACCACAGCCGCC	0.219	54	
SRSF2	c.284C>A	0.481	14	
SRSF2	c.284C>A	0.419	25	
SRSF2	c.284C>A	0.36	38	
SRSF2	c.284C>A	0.47	44	
SRSF2	c.284C>A	0.453	47	
SRSF2	c.284C>A	0.242	56	
SRSF2	c.284C>A	0.451	59	
SRSF2	c.284C>A	0.497	64	
SRSF2	c.284C>A	0.519	75	
SRSF2	c.284C>A	0.504	76	
SRSF2	c.284C>A	0.414	80	
SRSF2	c.284C>A	0.507	95	
SRSF2	c.284C>A	0.449	102	
SRSF2	c.284C>A	0.415	103	
SRSF2	c.284C>A	0.364	109	
SRSF2	c.284C>A	0.457	114	
SRSF2	c.284C>G	0.222	16	

Table S1A (Cont'd)

Gene	Variant	VAF	PN
SRSF2	c.284C>G	0.476	79
SRSF2	c.284C>T	0.399	7
SRSF2	c.284C>T	0.454	39
SRSF2	c.284C>T	0.468	43
SRSF2	c.284C>T	0.473	78
SRSF2	c.284C>T	0.348	82
SRSF2	c.284C>T	0.428	94
SRSF2	c.284C>T	0.255	106
STAG2	c.1536A>T	0.2	28
STAG2	c.1876dupA	0.11	25
STAG2	c.2536_2552delGGTCAGCAAGAGGATGA	0.373	25
STAG2	c.2562_2563insC	0.31	7
STAG2	c.913C>T	0.298	15
TET2	c.1803delC	0.491	79
TET2	c.1810C>T	0.51	100
TET2	c.1852C>T	0.344	24
TET2	c.1918C>T	0.315	92
TET2	c.2148dupA	0.493	72
TET2	c.2255_2261delATAAAGA	0.434	100
TET2	c.2311A>T	0.326	16
TET2	c.2382_2385delAAGC	0.431	106
TET2	c.2725C>T	0.454	92
TET2	c.2848_2849delGC	0.491	39
TET2	c.2905delC	0.441	102
TET2	c.2926C>T	0.341	67
TET2	c.2937delG	0.175	67
TET2	c.322C>T	0.387	48
TET2	c.3308delA	0.529	78
TET2	c.3365delC	0.387	102
TET2	c.3457G>A	0.188	61
TET2	c.3575delG	0.266	82
TET2	c.3782G>A	0.467	64
TET2	c.3811delT	0.268	89
TET2	c.3893delG	0.392	80
TET2	c.3922A>T	0.429	69
TET2	c.3965T>C	0.419	6
TET2	c.4075C>T	0.41	79
TET2	c.4393C>T	0.238	61
TET2	c.4662dupA	0.202	106
TET2	c.5029dupA	0.465	39
TET2	c.5081T>G	0.301	106
TET2	c.521delC	0.476	64
TET2	c.551_555delAGCAG	0.13	48
TET2	c.5541G>A	0.457	78
TET2	c.5650A>G	0.375	18
TET2	c.5650A>G	0.712	53
TET2	c.5686A>G	0.448	99
TP53	c.1129A>C	0.163	110
TP53	c.1129A>C	0.207	113
TP53	c.376-2A>G	0.318	9
TP53	c.376-8_384delTCCTACAGTACTCCCCT	0.122	90
TP53	c.427G>A	0.3	108
TP53	c.451C>T	0.806	84
TP53	c.470dupT	0.408	121
TP53	c.536A>G	0.321	42
TP53	c.615T>A	0.304	108
TP53	c.701A>C	0.091	97
TP53	c.743G>A	0.12	110
TP53	c.824G>A	0.17	105
TP53	c.830G>A	0.344	103
TP53	c.844C>T	0.893	40
U2AF1	c.470A>G	0.376	60
WT1	c.1137_1138insG	0.492	22
WT1	c.1137dupA	0.467	22
WT1	c.1141_1144dupTCGG	0.139	19
WT1	c.1397delT	0.231	71
ZRSR2	c.1207delA	0.222	73
ZRSR2	c.66delG	0.34	61

Table S1A. Pathogenic variants. Variants of uncertain significance reclassified as pathogenic by the custom onco-hematology score are highlighted in yellow. PN: patient number. ITD: internal tandem duplication. Onco-hematology score critical feature: * Remission sample. † VAF value.

Table S1B Variants of uncertain significance

Gene	Variant	VAF	PN
ABL1	c.1823G>A	0.467	24
ABL1	c.2066A>G	0.544	120
ANKRD26	c.3169G>A	0.501	114
ANKRD26	c.371A>T	0.502	109
ANKRD26	c.4145T>A	0.464	70
ANKRD26	c.4259G>A	0.453	119
ANKRD26	c.4924A>G	0.5	117
ANKRD26	c.542C>T	0.46	97
ASXL1	c.2898_2900delAGG	0.467	79
ASXL1	c.2911A>C	0.453	31
ASXL1	c.3306G>T	0.499	6
ASXL1	c.3306G>T	0.481	22
ASXL1	c.3449G>T	0.474	30
ASXL1	c.3745A>G	0.488	72
ASXL1	c.4189G>A	0.511	72
ASXL1	c.4493C>T	0.496	56
BCORL1	c.3108C>T	0.468	8
BCORL1	c.3302G>A	0.357	49
BRAF	c.1150A>G	0.442	20
CEBPa	c.296G>C	0.125	73
CNOT3	c.1136C>G	0.512	15
CNOT3	c.1277C>T	0.494	60
CNOT3	c.1528G>A	0.446	40
CREBBP	c.6077T>A	0.469	8
CREBBP	c.6624A>C	0.491	52
CSF3R	c.2153C>T	0.494	64
CSF3R	c.2153C>T	0.454	109
CSF3R	c.2197C>A	0.47	29
CSF3R	c.2405C>T	0.494	9
CSF3R	c.2422G>A	0.498	6
CSF3R	c.2422G>A	0.542	32
CSF3R	c.2503G>A	0.557	62
CSF3R	c.2503G>A	0.543	73
CSF3R	c.2503G>A	0.466	85
DDX41	c.1574G>A	0.114	97
DDX41	c.305A>G	0.485	112
DDX41	c.992_994delAGA	0.5	63
EPOR	c.137G>A	0.535	110
EPOR	c.296C>T	0.559	54
EPOR	c.368G>A	0.477	101
EPOR	c.971C>T	0.504	118
ETV6	c.1169C>T	0.222	66
ETV6	c.985G>A	0.448	107
EZH2	c.165C>G	0.445	57
FLT3	c.2440G>A	0.466	50
GNAS	c.1343A>C	0.156	61
KMT2A	c.10318A>G	0.367	40
KMT2A	c.10318A>G	0.501	66

Table S1B (Cont'd)

Gene	Variant	VAF	PN
KMT2A	c.10318A>G	0.437	97
KMT2A	c.1504G>A	0.396	86
KMT2A	c.1504G>A	0.495	87
KMT2A	c.3907C>G	0.496	69
KMT2A	c.3974G>A	0.503	39
KMT2A	c.6572G>A	0.478	21
KMT2A	c.6572G>A	0.417	32
KMT2A	c.6572G>A	0.48	67
KRAS	c.535G>A	0.35	71
KRAS	c.565A>C	0.446	92
KRAS	c.-9C>T	0.439	67
MAP1B	c.2768T>C	0.493	5
MAP1B	c.6077T>A	0.491	21
MPL	c.313T>C	0.522	81
MYD88	c.686T>C	0.486	60
NF1	c.1870T>C	0.521	48
NF1	c.4381A>T	0.224	75
NF1	c.528T>A	0.455	77
NOTCH1	c.1750G>A	0.481	12
NOTCH1	c.2734C>T	0.517	10
NOTCH1	c.4028C>T	0.5	4
NOTCH1	c.4865G>A	0.509	5
NOTCH1	c.5189C>T	0.523	33
NOTCH1	c.701G>A	0.488	53
PHF6	c.59G>A	0.364	49
RUNX1	c.493G>A	0.491	102
RUNX1	c.560C>T	0.508	32
SETBP1	c.1540C>G	0.48	112
SETBP1	c.3299A>G	0.493	69
SETBP1	c.3806A>G	0.173	96
SETBP1	c.3806A>G	0.275	97
SETBP1	c.3806A>G	0.269	102
SETBP1	c.3806A>G	0.217	116
SETBP1	c.3962G>A	0.481	44
SETBP1	c.3962G>A	0.46	104
SETD2	c.3422C>T	0.463	56
SETD2	c.5666T>C	0.438	7
SETDB1	c.2920G>A	0.515	43
SETDB1	c.2930C>T	0.479	32
SOS1	c.553A>G	0.552	22
TET2	c.3703_3704insTTC	0.433	99
TET2	c.4121G>A	0.476	45
TET2	c.4145A>G	0.395	61
TET2	c.5103G>T	0.448	63
TET2	c.5152G>T	0.499	26
TET2	c.5152G>T	0.499	50
TET2	c.541_543delATT	0.151	48
ZRSR2	c.1147C>G	0.539	41

Table S1B. Variants of uncertain significance. PN: patient number.

Table S1C. Benign variants

Gene	Variant	VAF	PN
ANKRD26	c.3655G>A	0.495	101
ANKRD26	c.3655G>A	0.398	110
ANKRD26	c.4445T>C	0.466	85
ANKRD26	c.4445T>C	0.47	116
BCOR	c.5102T>G	0.13	5
BCOR	c.5102T>G	0.136	60
CEBPa	c.584_589dupACCCGC	0.5	7
CEBPa	c.584_589dupACCCGC	1.0	11
CEBPa	c.584_589dupACCCGC	0.517	12
CEBPa	c.584_589dupACCCGC	0.492	13
CEBPa	c.584_589dupACCCGC	0.494	29
CEBPa	c.584_589dupACCCGC	0.454	37
CEBPa	c.584_589dupACCCGC	0.431	65
CEBPa	c.584_589dupACCCGC	0.397	99
CEBPa	c.584_589dupACCCGC	0.381	102
CNOT3	c.1414C>T	0.536	9
CREBBP	c.1651C>A	0.46	10
CREBBP	c.1651C>A	0.419	35
CSF3R	c.2197C>A	0.47	29
GNAS	c.1376C>G	0.391	2
KMT2A	c.89C>G	0.528	78
KMT2A	c.89C>G	0.486	91
MAP1B	c.2386G>A	0.491	13
NOTCH1	c.3836G>A	0.5	26
NOTCH1	c.4129C>T	0.463	5
NOTCH1	c.4129C>T	0.502	6
NOTCH1	c.4129C>T	0.625	42
NOTCH1	c.4129C>T	0.463	47
SETBP1	c.4129G>C	0.471	12
SETBP1	c.4129G>C	0.486	21
SETBP1	c.4129G>C	0.434	49
SETBP1	c.4599_4607delGCCGCCACC	0.451	59
SOS1	c.2122G>A	0.467	14
TET2	c.100C>T	0.475	8
TET2	c.100C>T	0.474	73
TET2	c.100C>T	0.467	84
TET2	c.2599T>C	0.528	96
TET2	c.2599T>C	0.504	104
TET2	c.5167C>T	0.473	96
TET2	c.5167C>T	0.476	104

Table S1C. Benign variants. PN: patient number.

Table S2. Germline variants

PN	Gene	Variant	VAF	VAF >0.4	Control sample	Germline*
1	*CEBPa*	c.944_945insCAC	0.392	No	-	No
1	*CEBPa*	c.247delC	0.418	Yes	Remission PB	No
6	*CEBPa*	c.178delA	0.496	Yes	Fibroblasts	No
7	*RUNX1*	c.611G>A	0.221	No	-	No
8	*RUNX1*	c.592G>A	0.363	No	-	No
9	*TP53*	c.376-2A>G	0.318	No	-	No
13	*CEBPa*	c.622T>C	0.5	Yes	Remission PB	No
13	*CEBPa*	c.659T>C	0.5	Yes	Remission PB	No
14	*RUNX1*	c.367_370dupGATG	0.307	No	-	No
16	*RUNX1*	c.926dupG	0.119	No	-	No
17	*GATA2*	c.1186C>T	0.491	Yes	Fibroblasts	Yes
19	*CEBPa*	c.68dupC	0.45	Yes	Remission PB	No
24	*GATA2*	c.913C>G	0.433	Yes	Fibroblasts	No
26	*CEBPa*	c.198_201dupCTAC	0.772	Yes	Fibroblasts	No
40	*TP53*	c.844C>T	0.893	Yes	Fibroblasts	Yes
42	*TP53*	c.536A>G	0.321	No	-	No
48	*RUNX1*	c.941_942delCT	0.229	No	-	No
51	*DDX41*	c.931C>T	0.517	Yes	Not available	-
55	*GATA2*	c.1187G>A	0.565	Yes	Fibroblasts	Yes
70	*CEBPa*	c.383delC	0.426	Yes	Remission PB	No
72	*CEBPa*	c.59_60insTC	0.478	Yes	Remission PB	No
73	*RUNX1*	c.595G>A	0.219	No	-	No
82	*RUNX1*	c.530_532delTCA	0.209	No	-	No
84	*TP53*	c.451C>T	0.806	Yes	Remission PB	No
89	*RUNX1*	c.335dupT	0.074	No	-	No
90	*TP53*	c.376-8_384delTCCTACAGTACTCCCCT	0.122	No	-	No
95	*RUNX1*	c.602G>A	0.379	No	-	No
97	*TP53*	c.701A>C	0.091	No	-	No
103	*ETV6*	c.416_419delCTAT	0.077	No	-	No
103	*TP53*	c.830G>A	0.344	No	-	No
105	*RUNX1*	c.941_942delCT	0.136	No	-	No
105	*TP53*	c.824G>A	0.17	No	-	No
108	*TP53*	c.615T>A	0.304	No	-	No
108	*TP53*	c.427G>A	0.3	No	-	No
109	*RUNX1*	c.305dupT	0.527	Yes	Not available	-
110	*TP53*	c.1129A>C	0.163	No	-	No
110	*TP53*	c.743G>A	0.12	No	-	No
112	*CEBPa*	c.971T>G	0.471	Yes	Fibroblasts	No
112	*CEBPa*	c.934_936dupCAG	0.49	Yes	Fibroblasts	No
112	*GATA2*	c.989G>A	0.412	Yes	Fibroblasts	No
113	*TP53*	c.1129A>C	0.207	No	-	No
116	*RUNX1*	c.958C>T	0.421	Yes	Remission PB	No
117	*ETV6*	c.306dupT	0.476	Yes	Not available	-
121	*TP53*	c.470dupT	0.408	Yes	Not available	-

Table S2. Germline variants. PN: patient number. PB: peripheral blood. *Confirmed in control sample.

Table S3

		Detection			
**PN**	**Alteration**	**NGS**	**Karyotype**	**FISH**	**qPCR**
9	del(5)	Yes	45,XY,del(2)(p14),**del(5)(q11)**,**del(7)(q22)**,-12,**der(17)**t(5;17)(q22;p13)[10] / 46,idem,r[cp2] / 46,XY[8]	Yes (43%)	-
	del(7)	Yes		Yes (48%)	-
	del(17p)	Yes		Yes (52%)	-
12	inv(16)	Yes	46,XY,**inv(16)(p13q22)**[18] / 46,XY[2]	NP	Yes
17	+8	Yes	47,XY,**+8**[20]	Yes (65%)	-
20	t(10;11)	Yes	47,X,t(Y;15)(q11;p11),**+8**,inv(12)(q13q15)[19]/46,XY[1]	Yes* (70%)	Yes
	+8	Yes		NP	-
46	del(7)**	No	No metaphases	Yes (90%)***	-
54	+8	No	47,XY,**+8**[7]/46,XY,der(22)t(1;22)(q11;p11)[5]/ 46,X,der(Y)t(Y;1)(q12;q11)[3]/ 46,XY,der(13)t(1;13)(q11;p11)[1]/46,XY,der(15)t(1;15)(q11;p11)[1]/ 46,XY[3]	NP	-
65	del(5)	Yes	46,XY,**del(5)(q13q33)**[3]/46,XY[17]	Yes (32%)	-
66	+8	Yes	47,XY,**+8**,del(12)(p12p13)[1]/47,XY,**+8**[13]/46,XY[6]	NP	-
68	del(20q)	Yes	46,XX,**del(20)(q12)**[3]/46,XX[6]	NP	-
76	+8	Yes	47,XY,**+8**[20]	NP	-
80	del(20q)	Yes	NP	Yes (83%)	-
82	+8	No	47,XX,**+8**[4]/46,XX[6]	NP	-
83	del(7)	Yes	45,XY,inv(3)(q21;q26),**-7**[15]/45,XY,inv(3)(q21;q26),**-7**,del(16)(q13)[3]/46,XY[2]	NP	-
84	del(5)	Yes	47~48,XY,**del(5)(q13q33)**,**-7**,**del(20)(q12)**,+mar1,dmin[cp10]/46,XY[4]	NP	-
	del(7)	Yes		NP	-
	del(20q)	Yes		NP	-
90	del(5q)	Yes	No metaphases	Yes (44%)	-
	del(7q)	Yes		Yes (42%)	-
	del(17p)	Yes		Yes (59%)	-
92	+8	No	50,XY,**+8**,del(9)(q13),+15,+20,+mar[cp10]/46,XY[1]	NP	-
95	+8	No	47, XY,**+8** [3],46,XY[10]	NP	-
98	del(7q)	Yes	46,XY,**del(7)(q31)**[16]/46,XY[4]	NP	-
102	+8	Yes	47,XY,**+8**[7]/46,XY[13]	NP	-
105	del(7)	Yes	45,XX,**-7**[1]/46,XX,**-7**,dmin[2]	NP	-
108	del(5q)	Yes	43,XX,add(1)(q32),add(5)(p13),**del(5)(q15q33)**,**-7**,del(9)(q22),-13,add(14)(p11),**-20** [17]/46,XX[3]	NP	-
	del(7)	Yes		NP	-
	del(20q)	Yes		NP	-
110	del(5q)	Yes	46,XY,**del(5)(q13q33)**[3]/47,XY,**del(5)(q13q33)**,+21[5]/46,XY[12]	NP	-
114	+8	Yes	47,XY,**+8**[20]	NP	-
116	+8	No	47,XX,**+8**[2]/46,XX[18]	NP	-
119	t(8;21)	Yes	45,X,-Y,**t(8;21)(q22;q22)**[8]/46,XY[12]	NP	Yes

Table S3. CNV and translocations. PN: patient number. NP: not performed. *KMT2A rearrangement break-apart probe. **Low infiltration. ***Performed in CD34+ isolated cells.

Table S4

Genes	Exons	Genes	Exons
GNB1	All	*WT1*	7 & 9
NRAS	2 & 3	*PTPN11*	3 & 13
SETDB1	All	*KRAS*	2 & 3
RIT1	All	*LTA4H*	All
FMN2	All	*FLT3-ITD*	14, 15 & 20
CSF3R	14 & 17	*POU4F1*	All
MPL	4, 10 & 12	*MAP2K1*	All
SF3B1	12 to 16	*AKAP13*	All
IDH1	4	*IDH2*	4
DNMT3A	All	*MAZ*	All
SOS1	All	*CREBBP*	All
RAF1	All	*DNAH9*	All
STAG1	All	*NF1*	All
MYD88	All	*PPM1D*	All
SETD2	All	*TP53*	All
TLR9	All	*SRSF2*	1
TET2	All	*SETBP1*	All
HTT	All	*CALR*	9
KIT	8 & 17	*CEBPA*	All
NPM1	12	*POU2F2*	All
MAP1B	All	*CNOT3*	All
CUX1	All	*ASXL1*	12 & 13
BRAF	All	*GNAS*	All
EZH2	All	*RUNX1*	3 to 8
NOTCH1	All	*U2AF1*	2 & 6
CA9	All	*STAG2*	All
JAK2	12, 14 & 15	*BCORL1*	All
SMC3	All	*ELF4*	All
SHOC2	All	*PHF6*	All
KMT2a	All	*ZRSR2*	All
CBL	8 & 9	*BCOR*	All

Table S4. Genes included in panel A.

Table S5

Genes	Exons	Genes	Exons
CSF3R	6 to 8 & 14 to 17	*KMT2A*	All
MPL	3 to 6, 10 &12	*CBL*	4, 5 & 8 to 11
NRAS	2 & 3	*ETV6*	All
DNMT3A	7 to 23	*ETNK1*	3
SF3B1	12 to 16	*KRAS*	2 to 5
IDH1	4	*PTPN11*	3, 4 & 13
VHL	2 & 3	*FLT3*	14, 15 & 20
GATA2	3 to 7	*IDH2*	4
KIT	8 & 17	*TP53*	2 to 11
TET2	All	*NF1*	All
NPM1	12	*SRSF2*	1
DDX41	All	*SETBP1*	4
IKZF1	2 to 8	*EPOR*	All
EZH2	All	*CALR*	9
JAK2	8 & 12 to 15	*CEBPa*	1
ABL1	4 to 11	*ASXL1*	12 & 13
ANKRD26	All	*RUNX1*	3 to 8
WT1	7 to 9	*ZRSR2*	All
**Translocations**		**Deletions**	
t(9;22)(q34;q11.2)		del4q12 (*CHIC2*)	
t(5;var)(q31-q33;var)		del 17p13.1 (*TP53*)	
t(8;var)(p11;var)		del(7q) [7q11-7q36]	
t(8;21)(q22;q22)		del(5q) [5q12-5q34]	
t(16;16)(p13.1;q22)//inv(16)(p13;q22)		del(11q)//amp(11q)	
t(17;var)(q21)		del(12p)	
t(11;var)(q23;var)		del(20q) [q11-qter]	
t(15;17)(q22;q21)		**Aneusomies**	
t(6;9)(p23;q34)		+8	
t(1;22)(p13;q13)		+19	

Table S5. Genes and alterations included in panel B.

Supplementary Information:

Variant Annotation

Variant transcript annotation was based on all human transcripts obtained from *Ensembl*, Release v81. Only variants located in the coding region and at the splicing sites of canonical isoforms were analyzed. Synonymous variants were excluded from the analysis. *Genecards* and *Uniprot* were used to obtain gene information about areas such as protein function, critical domains, and aliases. Population databases (*GenomAD* and *1000 genomes*) were used to determine the minor allele frequency (MAF) of each variant in order to identify and exclude polymorphisms (variants with MAF greater than 1% in the general population). *In silico* functional analysis was performed (*Mutation taster*, *Polyphen2*, *SIFT*, and *Human Splicing Finder*), and cancer-specific variant databases such as *COSMIC*, *Clinvar*, or *IARC TP53* were also verified. Finally, a thorough literature search was performed to collect data on the variant (eg, functional assays, case-control studies, and reports related to the disease).

Custom Onco-Hematology Score

The ACMG score [19] was modified in order to classify onco-hematologic somatic variants.

Two new features were added as supporting evidence of pathogenicity criterion (PP): VAF and absence in remission sample:

- ✓ VAF of the analyzed variant is considered as pathogenic criterion if it is similar to the VAF of other concomitant variants.

  Note: Variants with VAF values close to 0.5 cannot be evaluated (they could be benign variants).

- ✓ Absence of the variant in remission sample indicates somatic origin, and this could be associated with the disease.

Several characteristics were removed from the score since they were limited to pathogenicity evaluation of germline variants:

- ✓ "De novo (both maternity and paternity confirmed) in a patient with the disease and no family history" (Strong evidence of pathogenicity-PS).
- ✓ "For recessive disorders, detected in trans with a pathogenic variant" (Moderate evidence of pathogenicity-PM).
- ✓ " Assumed de novo, but without confirmation of paternity and maternity" (PM).

- ✓ "Cosegregation with disease in multiple affected family members in a gene definitively known to cause the disease" (PP).
- ✓ "Patient's phenotype or family history is highly specific for a disease with a single genetic etiology" (PP).

Validation

The presence of the variants selected was confirmed by PCR-based specific amplification and Sanger sequencing (*BigDye* v3.1; ABI3130xl or ABI3730xl, *Applied Biosystems*). The detection of internal tandem duplications (ITD) on the *FLT3* gene was validated by fluorescence-based fragment amplification and capillary electrophoresis (ABI3130xl or ABI3730xl, *Applied Biosystems*). Germline or somatic origin was tested in those patients with variants affecting genes associated with inherited myeloid neoplasms (genes underlined in figure 1) presenting a VAF higher than 0.4. The confirmation was performed by variant analysis in fibroblast culture and if not available, in a remission PB sample (Table S2). Cytogenetic alterations were confirmed through qPCR, FISH or/and karyotype. (Table S3).

Routine methodological approaches in myeloid neoplasm.

AML*:* *FLT3*-ITD, *NPM1* and *CEBPa* fragment analysis, *RUNX1/RUNX1T1*, *CBFB/MYH11* and *KMT2A/MLLT10* qPCR translocations, karyotype and FISH of *KMT2a* (break apart probe), inv3/t(3;3) and del(17p13) (Abbott Molecular, USA).

MDS: Karyotype

MPN and MDS/MPN: *JAK2* (V617F and if negative exon 12) for PV; and *JAK2*, *CALR* and *MPL* for PMF and ET were also considered conventional approaches.

In all MN, FISH [del(5q), -7, del(7q), del(20q) and +8] was also treated as conventional method in those patients with no metaphases.

List of Abbreviations

MN: Myeloid neoplasms

AML: Acute myeloid leukemia

MDS: Myelodysplastic syndrome

MPN: Myeloproliferative neoplasm

MDS/MPN: Myelodysplastic syndrome/myeloproliferative neoplasm

NGS: Next-generation sequencing

CNV: Copy number variants

VUS: Variants of uncertain significance

VAF: Variant allele frequency

PV: Polycythemia vera

PMF: Primary myelofibrosis

ET: Essential thrombocythemia

ITD: Internal tandem duplication

TKD: Tyrosine kinase domain

BM: Bone marrow

PB: Peripheral blood

NOS: Not otherwise specified

Allo-HSCT: Allogeneic hematopoietic stem cell transplant

ACMG: American College of Medical Genetics and Genomics

ELN: European leukemia net

NCCN: National comprehensive cancer network

WHO: World health organization

GIPSS: Genetically inspired prognostic scoring system
